# Effects of buprenorphine on model development in an adjuvant-induced monoarthritis rat model

**DOI:** 10.1371/journal.pone.0260356

**Published:** 2022-01-13

**Authors:** Mie S. Berke, Louise K. D. Fensholdt, Sara Hestehave, Otto Kalliokoski, Klas S. P. Abelson

**Affiliations:** 1 Dept. of Experimental Medicine, Faculty of Health and Medical Sciences, University of Copenhagen, Copenhagen, Denmark; 2 Dept. of Cell and Developmental Biology, University College London, London, United Kingdom; University of Arizona College of Medicine, UNITED STATES

## Abstract

Complete Freund’s adjuvant (CFA)-induced arthritis in rats is a common animal model for studying chronic inflammatory pain. However, modelling of the disease is associated with unnecessary pain and impaired animal wellbeing, particularly in the immediate post-induction phase. Few attempts have been made to counteract these adverse effects with analgesics. The present study investigated the effect of buprenorphine on animal welfare, pain-related behaviour and model-specific parameters during the disease progression in a rat model of CFA-induced monoarthritis. The aim was to reduce or eliminate unnecessary pain in this model, in order to improve animal welfare and to avoid suffering, without compromising the quality of the model. Twenty-four male Sprague Dawley rats were injected with 20 μl of CFA into the left tibio-tarsal joint to induce monoarthritis. Rats were treated with either buprenorphine or carprofen for 15 days during the disease development, and were compared to a saline-treated CFA-injected group or a negative control group. Measurements of welfare, pain-related behaviour and clinical model-specific parameters were collected. The study was terminated after 3 weeks, ending with a histopathologic analysis. Regardless of treatment, CFA-injected rats displayed mechanical hyperalgesia and developed severe histopathological changes associated with arthritis. However, no severe effects on general welfare were found at any time. Buprenorphine treatment reduced facial pain expression scores, improved mobility, stance and lameness scores and it did not supress the CFA-induced ankle swelling, contrary to carprofen. Although buprenorphine failed to demonstrate a robust analgesic effect on the mechanical hyperalgesia in this study, it did not interfere with the development of the intended pathology.

## Introduction

Animal models of arthritis have been used extensively for decades to understand the mechanisms of inflammatory arthritis and to screen for new therapeutic agents. While these models have the potential to provide important human benefits, experimental work with models of painful conditions are problematic from a welfare point of view [1]. Although pain management is imperative, analgesics are typically withheld [2]. The primary concern with using analgesia in experimental pain models is the risk of the analgesics confounding the disease model [3]. However, untreated pain is also problematic, since suffering and impaired welfare have negative effects on body function and can influence the experimental outcome as well [4–6]. If we wish to compromise neither animal welfare nor model validity, it is imperative to identify appropriate analgesic regimens that limit unnecessary pain, with minimal impact on the disease progression.

Several analgesics are directly unsuitable for use in inflammatory pain models–*e*.*g*. non-steroidal anti-inflammatory drugs—because they adversely influence the pathological development of arthritis and thereby affect the study objectives [7–10]. Appropriate analgesics must thus minimize the pain via other pharmacodynamic properties.

Buprenorphine is a semi-synthetic opioid that acts as a partial μ-opioid receptor agonist [11, 12]. It is a commonly used analgesic in laboratory rodents because of its long plasma half-life (6–12 hours), effective control of mild to moderate pain, its multiple administration routes and its relatively few adverse effects compared to other opioids [2, 11, 12]. In addition, buprenorphine has been reported to have less immunosuppressive properties than other opioids [13] and to decrease the corticosterone levels in rats [14, 15]. It is most often administered subcutaneously, but several other modes of delivery have been investigated, *e*.*g*. oral administration by gavage or voluntary ingestion [14, 16, 17]. Reported doses and duration of analgesic activity vary considerably. The recommended dose for rats ranges from 0.01 to 0.50 mg/kg, administered s.c. or i.v., and the suggested dosing intervals are reported anywhere between 5–12 hours [18–23].

A few studies have investigated the effect of analgesia on model development in pain models. Dougherty *et al*. [24] demonstrated that the use of lidocaine applied during nerve damage reduced the duration and magnitude of hyperalgesia in rats induced with partial constriction neuropathy, but not with partial transection neuropathy. Shankarappa *et al*. [25] reported that the onset of allodynia was delayed in rats where neuropathic pain was induced by spared nerve injury, if the animals were treated with liposomes containing saxitoxin and dexamethasone [25], while using the same model Hestehave *et al*. [26] demonstrated that the development of allodynia was not affected by a variety of peri-operative analgesic treatments with different mechanisms of action. In a complete Freund’s adjuvant (CFA) induced polyarthritis rat model, Walker and colleagues [27] showed that buprenorphine had no significant effect on disease progression assessed by joint swelling. Previous publications from our group have demonstrated the beneficial effects of post-surgical buprenorphine in rat and mouse models of cerebral ischemia, while not affecting the model-development [28, 29].

The present study aimed to study whether it is possible to minimize unnecessary pain in the induction stages of a rat model of CFA-induced monoarthritis. A recent study from our group [30] demonstrated that the period where the animal wellbeing is most affected in this particular model, is during the first three days after induction. Therefore, it was hypothesized that buprenorphine treatment, administered from induction and onward during model development, would have a positive effect on animal welfare and pain related behaviour. A group of animals treated with carprofen (a non-steroidal anti-inflammatory drug), served as a positive control, where reduction of joint stiffness and joint circumference was expected, contrary to buprenorphine treatment.

## Materials and methods

### Ethics statement

This study was approved by the Animal Experiment Inspectorate under the Danish Ministry of Environment and Food of Denmark (license number 2014-15-0201-00257). All experiments were carried out in an AAALAC accredited animal facility in accordance with the Guide for Care and Use of Laboratory Animals and with the European Union Directive 2010/63/EU.

### Animals and housing

Thirty-two male NTac:SD rats from Taconic (Ry, Denmark), weighing approximately 200 g on arrival, were used in this study. Inclusion of both sexes was not possible due to the size of the study, however this will be investigated in future studies. Animals were housed in pairs in type IV S individually ventilated cages (IVC) (size 480 x 375 x 210 mm) from Techniplast (Varese, Italy). The cages were lined with aspen chip bedding (Tapvei, Finland), and contained paper nesting materials (Lillico, United Kingdom), wooden sticks (Tapvei), polycarbonate rat retreats (Molytex, Denmark) and cardboard tunnels (Lillico) as environmental enrichment. The animals were kept in a room with a temperature of 22°C (± 2°C), a relative humidity of 55% (± 10%) and a 12 h light/dark cycle, with lights on between 6:00 and 18:00, with 15–30 min of twilight. The animals were provided with chow (Altromin 1314; Altromin GmbH & Co., Germany) and tap water *ad libitum*. Rats were allowed to habituate to the housing facilities for nine days before start of experimentation.

### Study design

Animals were randomly assigned into four experimental groups and housed in pairs receiving the same treatment (Table 1). The four groups consisted of: one negative control group with no intraarticular injection of CFA and with subcutaneous injections (s.c.) of saline instead of analgesic treatment (CTRL); one positive control group injected with CFA intraarticularly and with saline s.c. (CFA); and two experimental groups injected with CFA intraarticularly and treated with either buprenorphine s.c. twice daily (CFA + BUP) or carprofen s.c. once daily (CFA + CAR).

**Table 1:** Overview of control and experimental groups.

Groups	N	TTJ^a^ intraarticular injection	Treatment (s.c. injection)
Control (or CTRL)	8	-	Saline
Vehicle (or CFA)	8	CFA	Saline
Buprenorphine (or CFA + BUP)	8	CFA	Buprenorphine
Carprofen (or CFA + CAR)	8	CFA	Carprofen

^a^ TTJ refers to tibio-tarsal joint.

The induction of arthritis and the subsequent behavioural and analgesiometric testing were performed by the same observer, between 8:00 and 13:00, throughout the entire study, to avoid inter-observer differences. The treatment injections were performed by another person, and the behavioural observer was blinded to treatment groups. Pre-injury baseline measurements were performed the day before induction.

Group sizes were determined based on previous experience [30], and by a group size estimate using the resource equation method, as described by Mead (1988) and by Festing (2002 and 2003) [31–33], suggesting a number of six-to-eight experimental units per group.

### Induction of tibio-tarsal joint (TTJ) monoarthritis

Animals were briefly anesthetized with 3% isoflurane (“Attane Vet”, Isoflurane 1000 mg/g, ScanVet, Denmark) delivered in pure oxygen at a flow rate of 1.0 L/min using an induction chamber. When loss of righting reflex occurred, the anaesthetized rat was moved to a face mask. The rat was placed in right lateral recumbency and absence of pedal withdrawal reflex was confirmed before injection. Complete Freund’s adjuvant (CFA) (Sigma-Aldrich, USA), containing heat-killed and dried *Mycobacterium tuberculosis* (strain H37Ra, ATCC 25177), was injected (20 μl) into the left TTJ. The animal was then moved to their homecage for recovery. The entire procedure from induction to recovery was less than 10 minutes for each individual animal. Injections of pre-emptive analgesic or saline were administered 60 min before injection of CFA. Negative controls were subjected to anaesthesia alone, without intraarticular injection.

### Analgesic treatment

The analgesic-treatment consisted of either buprenorphine (“Temgesic” 0.3 mg/ml; RB Pharmaceuticals Ltd, UK) or carprofen (“Rimadyl vet.” 50 mg/ml; Pfizer Inc., USA). Treatment was initiated pre-emptively on day 0, followed by repeated daily injections until day 15, 60 min prior to behavioural testing. Doses were based on published recommendations [34–38]. Buprenorphine was administered subcutaneously (0.05 mg/kg) 8:00 and 16:00. Carprofen was administered subcutaneously (5 mg/kg daily) at 8:00 and were injected secondary with saline 16:00. The drugs were diluted in isotonic (0.9%) saline to provide an injection volume of 1 ml/kg s.c. of buprenorphine or carprofen. Rats that did not receive analgesia, received 1 ml/kg of isotonic saline subcutaneously at 8:00 and 16:00.

### Welfare assessment

All animals were subjected to a daily welfare assessment (WA) while in their home cages. The WA was performed as previously described [30, 39]. General appearance, porphyrin staining, gait and posture, wounds and body weight changes were assessed (Table 2). Scores of all parameters were pooled and used for determining welfare impairment and whether humane endpoints were reached. If the pooled score exceeded 0.4, the animal would be euthanized. No animals in this study exceeded this threshold.

**Table 2:** Welfare assessment.

General appearance	Reference score
Bright and alert	0
Burrowing or hiding, quiet but rouses when touched	0.1
Burrowing or hiding, quiet but rouses when touched. No exploration when lid off, burrows, hides, head presses. Might be aggressive when touched	0.4
Porphyrin staining	
None	0
Mild	0.1
Obvious on face or paws	0.4
Gait and posture	
Normal	0
Mild incoordination when stimulated, hunched posture, mild piloerection	0.1
Obvious ataxia or head tilt, hunching, severe piloerection	0.4
Body weight loss from baseline (pre-CFA-injection)	
<5%	0
5–10%	0.1
10–20%	0.4
Self-injury	
None	0
Bites or scratches itself, leading to wounds	0.4

Welfare assessment score sheet adapted from Hampshire et al., 2001 [39].

### Model-specific parameters

Scoring of arthritis was performed as previously [30], assessing mobility, stance, lameness and stiffness of the joint (Table 3). Mobility, stance and ambulation impairment were assessed at pre-injury baseline and on days 0–22 post injury, and stiffness scores were assessed at pre-injury baseline and on days 1, 3, 6, 9, 13, 16 and 20.

**Table 3:** Model specific parameters.

Mobility	Reference score
The rat walks and runs normally	0
The rat walks and runs with difficulty	1
The rat walks with difficulty	2
The rat crawls using front legs only	3
The rat lies down only	4
Stance	
The rat stands bearing weight equally on all four limbs	0
The rat stands bearing some weight on the arthritic limb	1
The rat stands with the arthritic paw touching floor, toes curled under	2
The rat stands on three paws only	3
Lameness	
Normal ambulation	0
Mild, slight lameness	1
Moderate, toe touching ground	2
Severe, limb carried	3
Joint stiffness	
Normal	0
Restriction of full range of flexion or extension	1
Restriction of full range of flexion and extension	2

Model specific parameter score sheet modified from Butler et al., 1992 [40].

The joint circumferences were assessed at pre-injury baseline and on days 1, 3, 6, 9, 13, 16 and 20 post injury. The circumferences were estimated by measuring perpendicular diameters of the joint using digital calipers. The circumference (C) was calculated as $\;C=2\;\times \;\pi \;\times \;\sqrt{0,5\;\times \;\left(a^{2}+b^{2}\right)}$, where ‘a’ was the radius of the dorso-plantar axis and b was the radius of the medio-lateral axis.

### Electronic von Frey test

An electronic von Frey (EVF) device (Model EVF3 with a hard tip, Bioseb, France) was used to assess mechanically evoked hyperalgesia. Assessments were made at pre-injury baseline and on days 1, 3, 6, 9, 13, 16 and 20. Rats were placed on a raised metal grid platform in acrylic chambers (size 16.5 x 24.2 x 14.6 cm) for 30 minutes of habituation before testing. Mechanical hyperalgesia (secondary mechanical hyperalgesia) was evaluated by applying a rigid plastic monofilament to the plantar surface of the paw, nearest the TTJ, with increasing force. The applied weight in gram (g) required to elicit paw withdrawal was recorded as the mechanical threshold. The test was repeated three times for each hind paw, starting with the non-injured right paw. The EVF tip was only applied when the rat stood still in a natural position with all four paws placed on the grid and a paw withdrawal response was only recorded with a complete lifting of the stimulated hind paw.

### Rat grimace scale

In an attempt to quantify spontaneous pain-related behaviour, the Rat Grimace Scale (RGS) was applied [41]. All animals were habituated to the observer and the observation method for six days prior to scoring. RGS was assessed pre-injury for baseline values and then on days 0–21 post injury. The blinded scoring of facial expressions was done while the rats were in their home cages, after the lid was carefully removed and after a habituation time of approximately 3 minutes. The scoring was done as a single point real-time observation [42] of approximately 15 seconds. Four action units (orbital tightening, ear changes, nose/cheek flattening and whisker changes) scored: “0” if not present, “1” if moderately present, “2” if obviously present, were used to produce an average score between 0–2 for each animal [41]. Scoring was not performed if the animal was rearing, sniffing, grooming or sleeping.

### Measurement of faecal corticosterone metabolites

Faeces were collected from all animals, pre-injury, and on days 1, 8, 15 and 20, in an attempt to non-invasively identify the severity of nociception-induced stress through quantitating excreted corticosterone and corticosterone metabolites. The time points were selected as they were considered to reflect acute, intermediate and late stages respectively, of the development of model parameters. The procedure was similar to what has previously been published from our group [43, 44]. Before faecal sample collection, the rats were placed in clean cages. Twenty-four hours later, all bedding and faeces were collected from each cage (each sample representing two rats of the same treatment group) and the faecal pellets were separated from the bedding by hand. The faecal samples were placed in plastic re-sealable bags and stored at -20 °C until analysis. For the analysis, faecal pellets were weighed and mixed with ethanol (5 ml/ g solid matter). Samples were incubated on a shaking table in 50 ml tubes for approximately 12 hours for extraction of faecal corticosterone and corticosterone metabolites (further referred to as FCM). The suspension was centrifuged at 2000 x g (Hermle Labortechnik GmbH, Germany) for 20 minutes, where after the supernatant was collected and the pellet discarded. One ml of the supernatant was further centrifuged at 10,000 x g for 15 minutes (Eppendorf 5415D; Eppendorf AG, Hamburg, Germany). The extracted sample was then evaporated to dryness under vacuum (Genevac EZ-2 personal evaporator, SP Scientific, USA) and resuspended in 0.15 M phosphate buffered saline (PBS, pH 7.2). The sample was again centrifuged at 10,000 x g and the clear liquid was analysed by ELISA (DRG-Diagnostics corticosterone competitive enzyme-linked immunosorbent assay), according to the manufacturer’s instructions.

### Histopathology

All rats were euthanized on day 23 by decapitation. Both hindlegs were collected immediately after euthanasia by a bilateral dissection proximal to the knee. The hind legs were placed in 50 ml containers and decalcicifed for 48 h in Osteomoll (Sigma-Aldrich). The ankles were trimmed according to Bolon *et al*. 2011 using a blade/scalpel [45]. The tissues were then dehydrated in 70% ethanol, and finally 90% ethanol before paraffin embedding and sectioning with a microtome. Sections were mounted on glass slides, and hematoxylin-eosin (HE) stain was applied. Detection of degenerative-inflammatory changes was performed blinded using an Olympus BX53 microscope (Olympus Corporations, Japan), and representative pictures were taken using infinityx-32c-Deltap SN 0174267 (Lumenera Corporations, Canada).

### Statistics

The statistical analysis was performed using GraphPad Prism version 8.0. Data were tested for normality using D’Agostino and Pearson omnibus normality test. Some values were missing in BW, circumference and stiffness because of technical mistakes, why a repeated-measures mixed-effects model analysis (using restricted maximum likelihood (REML) estimation) was applied for BW and circumference. Electronic von Frey and FCM were analysed by two-way repeated-measures (RM) ANOVA followed by a Tukey’s multiple comparisons test. FCM pre -and post-CFA-injection comparisons were analysed by two-way RM ANOVA with Bonferroni’s multiple comparisons test. Mobility, stance, stiffness and lameness scores were analysed by one-way non-parametric ANOVA (Kruskal-Wallis test) followed by Dunn’s multiple comparisons test. RGS data were analysed by one-way non-parametric ANOVA (Kruskal-Wallis test) followed by Dunn’s multiple comparisons test for each testing time, and by an ordinary one-way ANOVA followed by Tukey’s multiple comparisons test when expressed as AUC (area under the curve) values. p < 0.05 was considered statistically significant.

## Results

### General welfare assessment

**Fig 1:**
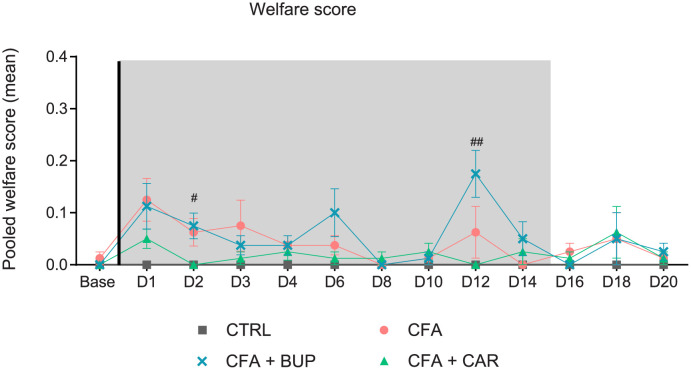
Welfare assessment (WA) scores. The figure shows pooled mean WA scores collected daily with the score sheet shown in Table 2. Statistics are calculated on ranks (Kruskal-Wallis test, followed by Dunn’s multiple comparisons test), but values are displayed as means ± SEM for ease of viewing. The hash sign (^#^) represents a statistically significant difference between CFA + BUP and CFA + CAR at ^#^p < 0.05 and ^##^p < 0.01. For all graphs: base = baseline values, D = day, the line represents the day of analgesic treatment initiation (D0) and the grey area represents the treatment period (15 days). All groups, *n =* 8, male Sprague Dawley rats.

All CFA-injected rats developed typical clinical symptoms of severe inflammation (erythema and edema of the CFA-injected ankle) observed 8 h after CFA injection and with a rapid progression. Besides the obvious inflammatory reaction, the rats’ welfare was not considerably affected. The pooled scores shown in Fig 1 did not exceed 0.4, *i*.*e*. the humane endpoint, for any of the subjects. Kruskal-Wallis tests detected significant higher scores in CFA + BUP compared to CFA + CAR day 2 (*H* (3) = 6.848, *p* = 0.0326) and day 12 (*H* (3) = 9.840, *p* = 0.0073) but did not differ from the CFA group. Mild porphyrin staining around eyes and nose was the most numerous indicator of impaired wellbeing in all CFA-injected animals.

**Fig 2:**
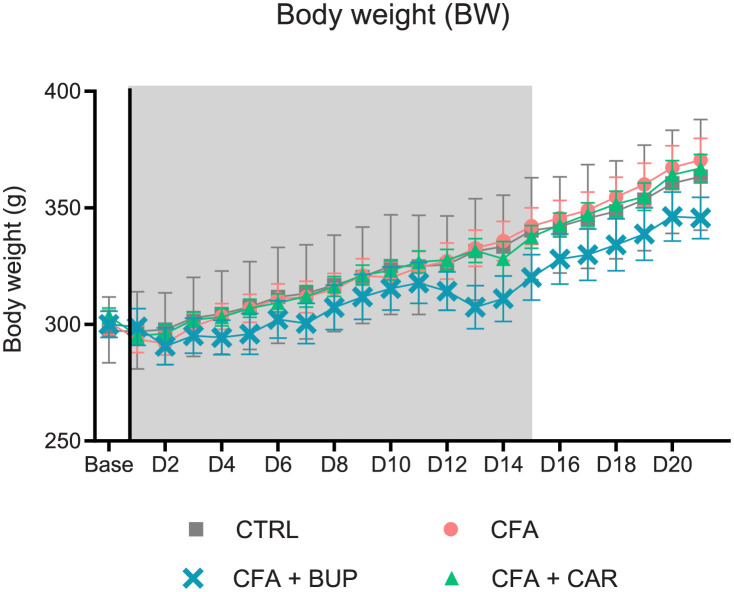
Body weight (BW). Mean body weight per group on day 1–22 and baseline values. Data were analysed by a two-way RM ANOVA followed by Tukey’s multiple comparisons tests. The line represents the day of analgesic treatment initiation (D0) and the grey area represents the days with treatment administrated. Values are expressed as mean ± SEM. For all graphs: base = baseline values, D = day, the line represents the day of analgesic treatment initiation (D0) and the grey area represents the treatment period (15 days). All groups: *n* = 8, male Sprague Dawley rats.

The body weight (BW) is shown in Fig 2. Mixed-effects model (REML) revealed a significant change over time (*F* (2.941, 82.20) = 441.4, *p* <0.0001) and a significant interaction between treatment and time (*F* (63, 587) = 4.169, *p* <0.0001). There were no significant differences in BW day 1 after CFA injection although a visible trend towards a decrease could be observed, continuing to day 2. A few of the rats suffered a weight loss of almost 10% of their baseline weight on day 1 (max-min individual weight loss range: CFA: 22–2 g, CFA + CAR: 14–5 g and CFA + BUP: 38–0 g). The CTRL group did not display either weight loss or gain day 1. From day 2 and throughout the study, the BW increased steadily in all groups. The CFA + BUP group demonstrated a noticeable body weight loss around day 13, however, no statistically significant differences between groups were detected at any time.

### Model-specific parameters

**Fig 3:**
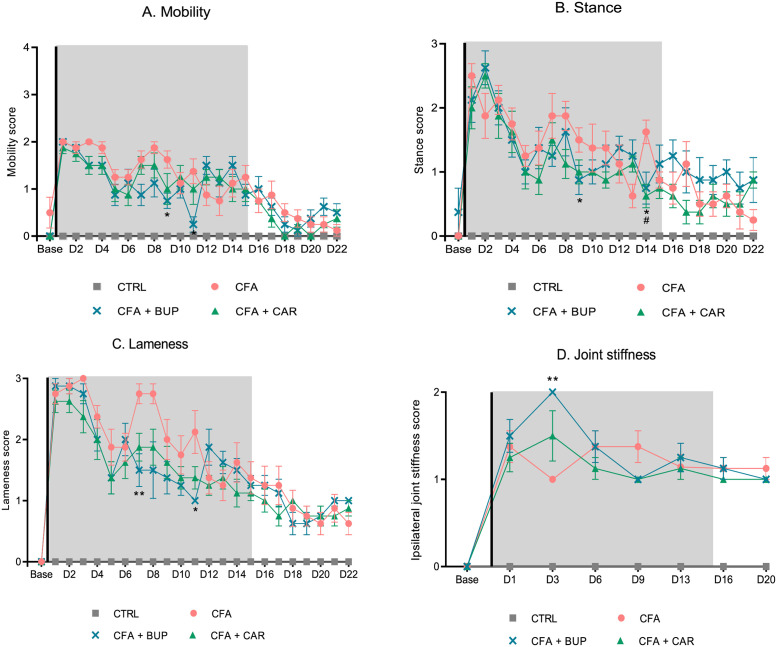
Model-specific parameters. **(A)** Mobility scores. **(B)** Stance scores. **(C)** Lameness scores. (**D)** The joint stiffness score of the ipsilateral TTJ. Statistics are calculated on medians (Kruskal-Wallis test, followed by Dunn’s multiple comparisons test), but values are displayed as mean ± SEM for a better graphical view. The asterisk (*) represents statistically significant difference between CFA + BUP and CFA at *p < 0.05 and **p < 0.01. The hash sign (^#^) represents a statistically significant difference between CFA + CAR and CFA at ^#^p < 0.05. For all graphs: base = baseline values, D = day, the line represents the day of analgesic treatment initiation (D0) and the grey area represents the treatment period (15 days). All groups, *n* = 8, male Sprague Dawley rats.

Mobility, stance and lameness scores are illustrated in Fig 3(A)–3(C). All CFA groups showed statistically significant differences in scores compared to baseline with the biggest difference within day 1–4 after CFA injection followed by a trend approaching the baseline scores as the study progressed. A Kruskal-Wallis test detected no statistically significant differences between CFA-injected groups on these parameters within the first week after CFA-injection. However, after the first week, the CFA + BUP group demonstrated a statistically significant difference from the CFA group in mobility (Fig 3A) day 9 (*H* (3) = 6.112, *p* = 0.0471) and day 11 (*H* (3) = 7.303, *p* = 0.0259). Similarly, differences were detected in the same period for the stance score (Fig 3B) day 9 (*H* (3) = 6.986, *p* = 0.0307) and day 14 (*H* (3) = 11.01, *p* = 0.0041), and for the lameness score (Fig 3C) day 7 (*H* (3) = 10.15, *p* = 0.0062) and day 11 (*H* (3) = 7.849, *p* = 0.0198). The CFA + CAR group demonstrated a significant difference in stance scores compared to the CFA group, day 14 (*H* (3) = 11.01, *p* = 0.0041).

The joint stiffness scores are presented in Fig 3D. All CFA-injected animals manifested stiffness of the joint in either flexion or extension, peaking on days 1–3, with a subsequent plateau. On day 3, the CFA + BUP group was significantly different from the CFA group (*H* (3) = 9.208, *p* = 0.0043), suggesting more stiffness of the joint on this particular day, but besides this, there were no significant differences between the CFA-injected groups.

### Joint circumference

**Fig 4:**
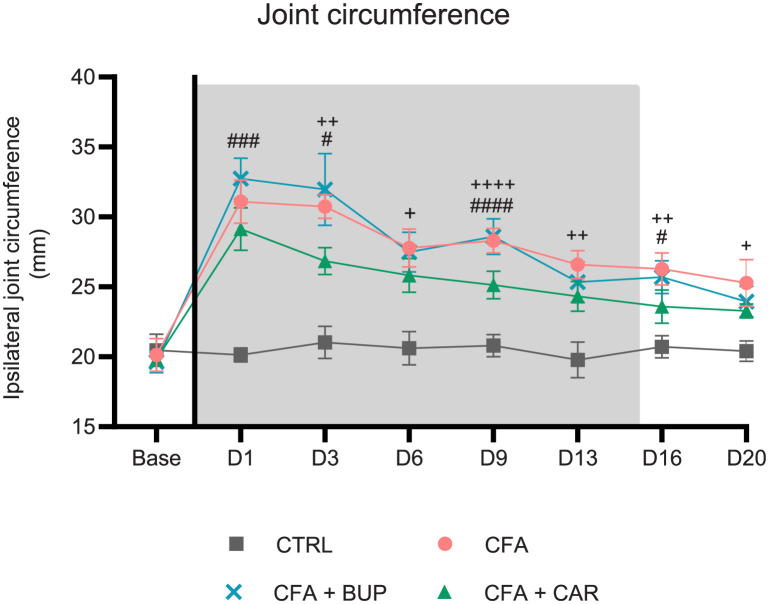
Circumference measurements of the ipsilateral ankle. The plus (^+^) represents a statistically significant difference between CFA + CAR and CFA at ^+^p < 0.05, ^++^p < 0.01 and ^++++^p < 0.0001. The hash (^#^) represents statistically significant difference between CFA + BUP and CFA + CAR at ^#^p < 0.05, ^###^p < 0.001 and ^####^p < 0.0001. For all graphs: base = baseline values, D = day, the line represents the day of analgesic treatment initiation (D0) and the grey area represents the treatment period (15 days). For all data: presented as mean ± SEM. All groups, *n* = 8, male Sprague Dawley rats.

Circumferences of the ipsilateral ankle are presented in Fig 4. A mixed-effects model (REML) revealed a significant difference between treatments (*F* (3, 28) = 138.8, *p* < 0.0001), over time (*F* (4.952, 128.8) = 209.2, *p* < 0.0001) and a significant interaction between treatment and time (*F* (21, 182) = 27.74, *p* < 0.0001). A significant increase in circumference was present in all CFA-injected groups compared to the CTRL group (day 1–20), with a peak for all CFA-injected groups on day 1 (*p* < 0.0001). The CFA + CAR group demonstrated a significantly lower circumference compared to the CFA group on day 3 (*p* < 0.01), day 6 (*p* < 0.05), day 9 (*p* < 0.0001), day 13 (*p* < 0.01), day 16 (*p* < 0.01) and day 20 (*p* < 0.05). Likewise, the CFA + CAR group differed significantly with a lower circumference from the CFA + BUP at day 1 (*p* < 0.001), day 3 (*p* < 0.05), day 9 (*p* < 0.001) and day 16 (*p* < 0.05). No significant differences were found between CFA + BUP and CFA at any time (Tukey’s multiple comparisons test).

### Assessment of pain-related behaviour

**Fig 5:**
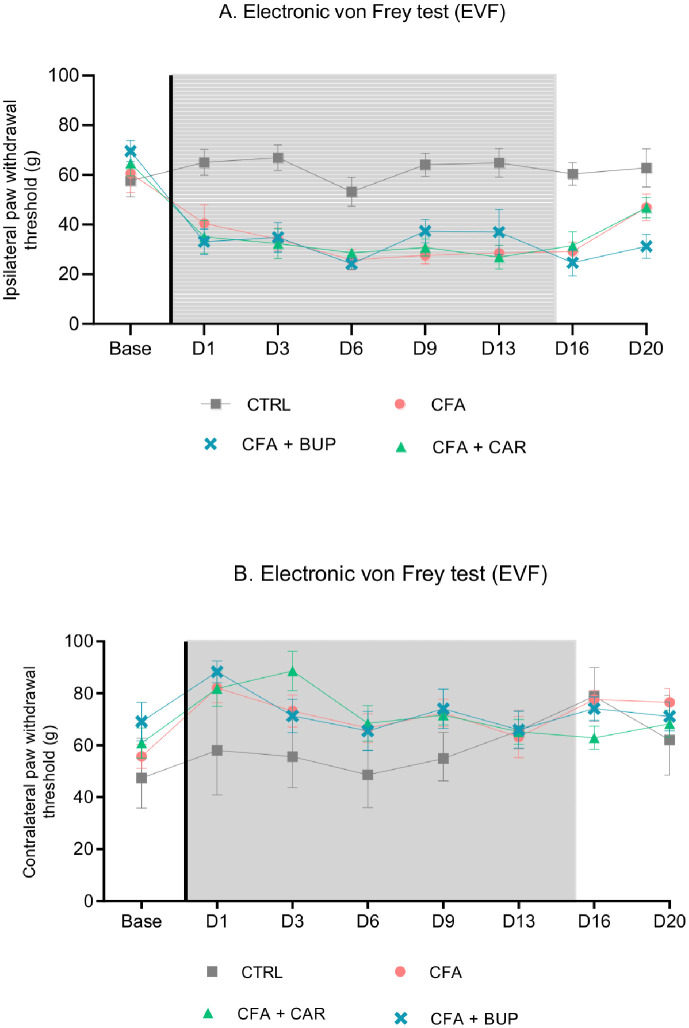
Assessment of mechanical threshold. Effects of buprenorphine and carprofen on paw withdrawal thresholds (g) as an expression of mechanical hyperalgesia. **(A)** Ipsilateral (left, CFA-injected paw) paw withdrawal thresholds. **(B)** Contralateral (right, non-injected paw) paw withdrawal thresholds. Data are analysed by a two-way RM ANOVA followed by a Tukey’s multiple comparisons test D1, D3, D6, D9, D13, D20 and presented as mean ± SEM. For all graphs: base = baseline values, D = day, the line represents the day of analgesic treatment initiation (D0) and the grey area represents the treatment period (15 days). All groups: *n* = 8, male Sprague Dawley rats.

The results of EVF testing on the ipsilateral paw are illustrated in Fig 5A. Two-way RM ANOVA revealed a significant difference between treatments (*F* (3, 28) = 22.08, *p* < 0.0001), over time (*F* (5.372, 150.4) = 13.69, *p* < 0.0001) and a significant interaction between treatment and time (*F* (21, 196) = 2.67, *p* = 0.0002). All CFA-injected groups had a significantly lower mechanical threshold day 1–16 compared to the CTRL group, indicating mechanical hyperalgesia secondary to the ankle-joint injury. No differences were detected between CFA-injected groups at any time (Tukey’s multiple comparisons test). Occasionally exaggerated responses were noticed after stimulation such as paw-shaking, paw grooming and guarding of the ipsilateral hind limb. On the contralateral paw (Fig 5B), statistically significant differences were seen between treatment (*F* (3, 28) = 3.899, *p* = 0.0191) and over time (*F* (5.377, 150.6) = 5.115, *p* = 0.0002). Tukey’s multiple comparisons test detected statistically significant differences between the CTRL group day 3 (CFA + CAR *p* = 0.0448) and day 9 (CFA *p* = 0.0366) and CFA + CAR (*p* = 0.0158).

**Fig 6:**
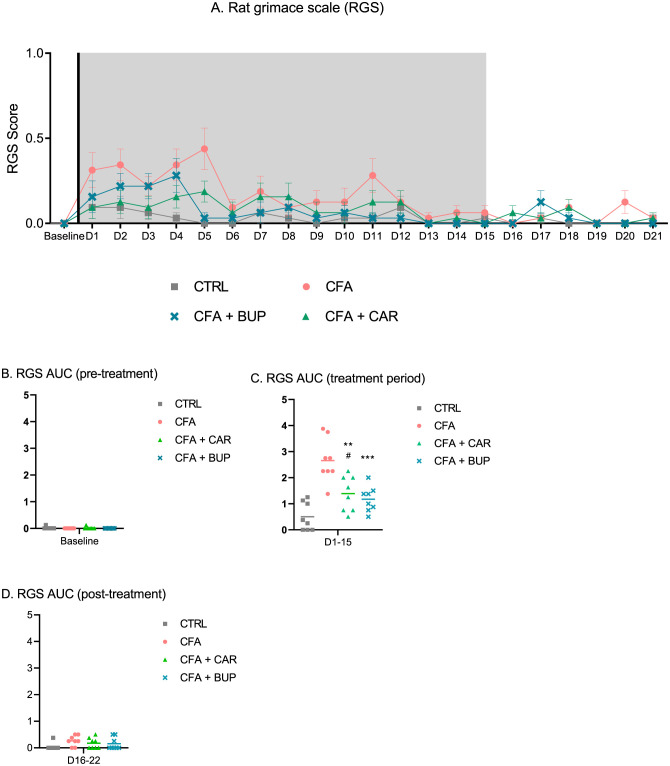
Assessment of the rat grimace scale (RGS). Facial expressions as a measure of non-evoked pain. Scoring was performed every 24 hours for 21 days with six baseline scorings before induction (D-6 to D0). **(A)** RGS scores at baseline (D-1) to D21. The line represents the day of analgesic treatment initiation (D0) and the grey area represents the treatment period (15 days). **(B)** Area under the curve (AUC) for baseline (pre-treatment) RGS scores, presented as a scatter plot. **(C)** AUC for D1-15 (treatment period), presented as a scatter plot. **(D)** AUC for D16-21 (post-treatment), presented as a scatter plot. The asterisk (*) represents statistically significant difference from the CFA group at **p < 0.001 and ***p < 0.0001. The hash sign (^#^) represents statistically significant difference between CFA + BUP and CFA + CAR at ^#^p < 0.05. For all graphs: base = baseline values, D = day, the line represents the day of analgesic treatment initiation (D0) and the grey area represents the treatment period (15 days). For all data: presented as mean ± SEM. For all groups: *n* = 8, male Sprague Dawley rats.

All mean RGS scores are presented in Fig 6. Scores were below 1 throughout, with the overall highest RGS scores detected within the first week, in particular in the CFA group. No statistically significant differences were detected between treatment groups at any time point analysed by Kruskal-Wallis tests (Fig 6A). But when comparing the RGS scores as AUC values for three periods—pre-treatment (baseline, Fig 6B), treatment (day 1–15, Fig 6C) and post-treatment (day 16–22, Fig 6D)—a one-way ANOVA revealed a significant difference in treatment (*F* (3, 28) = 16.60, *p* < 0.0001) during the treatment period. No significant differences were found pre-treatment or post-treatment. In the treatment period, Tukey’s multiple comparisons test detected significant differences between CFA vs. CFA + CAR (*p* = 0.0027) and CFA + BUP (*p* = 0.0005) and CTRL (*p* < 0.0001). Additionally, CFA + CAR had significantly higher RGS scores compared to CTRL (*p* = 0.0468), whereas no significant differences were detected between CFA + BUP and CTRL.

### Faecal corticosterone quantitation

**Fig 7:**
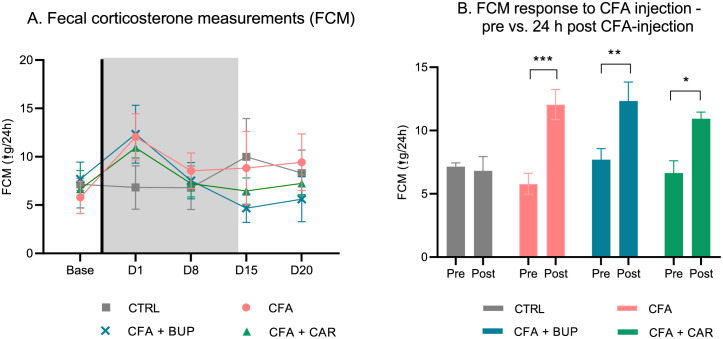
Faecal corticosterone measurements. **(A)** Faecal corticosterone metabolites (FCM) measurements are displayed as μg/24 h at baseline and D2, D9, D16 and D21. **(B)** Pre (baseline measurements) -and post-CFA-injection (D2 measurements) are compared. The asterisk represents statistical significant difference at *p < 0.05, **p < 0.01 and ***p < 0.001. For all data: presented as mean ± SEM. For all graphs: base = baseline values, D = day, the line represents the day of analgesic treatment initiation (D0) and the grey area represents the treatment period (15 days). For all groups: *n* = 8, male Sprague Dawley rats.

Average values of faecal corticosterone metabolites (μg) excreted during 24 hours per cage is presented in Fig 7. A mixed-effects model revealed a significant difference in time (*F* (2.603, 30.58) = 8.343, *p* = 0.0006) and a significant interaction between treatment and time (*F* (12, 47) = 3.489, *p* = 0.0010). Significant increases in corticosterone levels were observed in all CFA-injected groups on day 1 compared to baseline (CFA group (*p* = 0.0007), CFA + BUP (*p* = 0.0071) and CFA + CAR group (*p* = 0.0123)) (Fig 7B, Sidak’s multiple comparisons test), but not in the control group. No differences were found between treatment groups at any time point.

### Histopathological assessment

**Fig 8:**
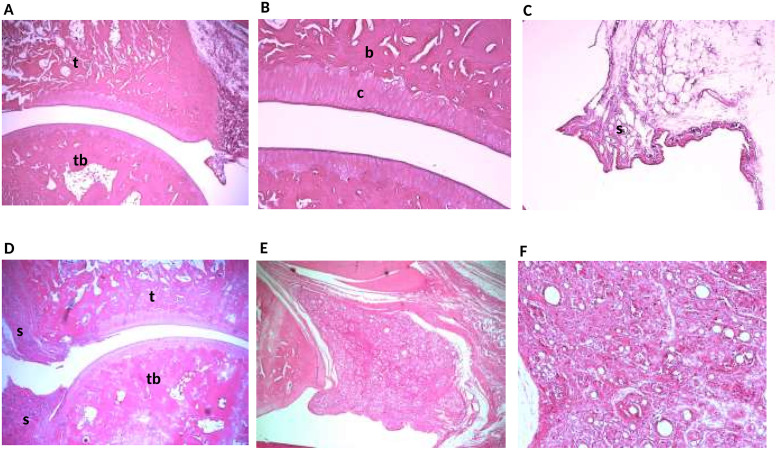
Hematoxylin-eosin (HE) sections. **A**. Normal articular spacing between tibia and the tarsal bone with smooth articular surface and a normal synovial lining (magnification 4x). **B**. Normal chondrocytes in columnar orientation and intact subchondral bone (magnification 4x). **C**. Normal synovial lining structures (Intima and subintima). Intima made up by 1–3 cells (synoviocytes) and a relatively loose connective tissue layer below with blood vessels and adipose cells (magnification 10x). **D**. Heavily cell-infiltrated tissue invading cranially and caudally into the tibio-tarsal joint causing local ulcerations in the articular cartilage and subchondral bone tissue (magnification 4x). **E**. Massive inflamed granulation tissue in the subintimal region together with synovial membrane hyperplasia (magnification 10x). **F**. Cells in the granulation tissue (macrophages, lymphocytes and plasma cells) surrounding lipid droplets of CFA in varying sizes (magnification 20x). In the pictures: b, bone; c, cartilage; s, synovial tissue; t, tibia; tb, tarsal bone.

The histopathological changes are presented in a descriptive manner, as a histological scoring process to depict differences between groups was shown not to be reliable. Representative histological sections are shown in Fig 8. The tibio-tarsal joints from control animals (Fig 8A–8C) showed a normal joint space, normal synovial lining and intact articular cartilage and subchondral bone tissue. In contrast, the tibio-tarsal joints of CFA-injected animals (Fig 8D–8F) revealed massive intra–and periarticular lesions. In the subintimal region inflamed granulation tissue dominated, making the synovium hypertrophic and the synovial lining hyperplastic. The inflammatory cells in the granulation tissue were identified as macrophages, lymphocytes and plasma cells, which were surrounding remnants of lipid droplets of CFA present in varying size. Within the joint space, histiocytic granulation tissue (pannus) was invading from the capsular angle causing focal erosion/ulcerations in the articular cartilage and subchondral bone tissue. Adjacent joints (the talo-navicular and talo-calcaneal joints) were found to be affected as well, with some destruction of articular cartilage and subchondral bone by the formation of similar inflamed tissue.

## Discussion

This study examined the effects of buprenorphine treatment on welfare, model-specific parameters and pain-related behaviour in a rat model of monoarthritis. The aim was to investigate whether it would be possible to minimize unnecessary pain related to the induction of this model, without compromising the quality of the model.

In general, animal welfare was not significantly affected throughout the study, as determined by the welfare assessment protocol (Hampshire et. al. [39]) (Table 2). However, data revealed two periods where welfare was affected—the first days after CFA injection (day 1–6), with clear signs of acute inflammation and pain-related behavior, and a less obvious chronic phase starting from day 10–12, lasting until termination on day 21, aligning with a previous study from our group [30].

In the first two days after CFA-injection, all rats demonstrated a lack of appropriate daily weight gain presumably due to loss of appetite as a sequela to anesthesia. The buprenorphine group stood out with a weight loss trend from day 12. Although this could indicate a decrease in general welfare, several studies have reported reduced food intake as a result of loss of appetite or metabolic alterations in response to buprenorphine administration [26, 46, 47], and this is likely the explanation for the decrease in BW and subsequently greater impairment of welfare scores in the current study as well.

Several model-specific parameters such as lameness, joint stiffness, stance and mobility were affected, as expected. Overall, significant differences in arthritis related parameters were observed between CFA-injected groups. Primarily in the buprenorphine group, a reduced degree of impairment from the injury was observed. The buprenorphine group and vehicle group had similar circumferences of the CFA injected joint, whereas the carprofen group showed a reduction of circumference almost from the first testing point after CFA injection and throughout the study. The reduction in joint circumference was expected because of carprofen’s anti-inflammatory effects [48]. The comparable joint circumferences in the buprenorphine and vehicle groups indicate that buprenorphine, in contrast to carprofen, did not suppress the inflammatory swelling in this model, as hypothesized.

Unexpectedly, no analgesic effect of buprenorphine could be detected in the EVF test, neither during the first three days after CFA-injection, where the inflammation and pain-related behaviour peaked, nor later in the treatment period. This may be explained by the dose of the drug being too low or the dosing interval being too infrequent, calling for further investigation of treatment protocols with buprenorphine. In addition, studying the first three days after induction more closely with more frequent intervals of assessment, may provide more robust data.

Mechanical allodynia, a painful response to a normally non-painful stimulus, is traditionally measured by applying low-intensity stimuli, such as prodding the skin with thin bending von Frey filaments, while hyperalgesia is exaggerated sensitivity to stimuli, which would also be considered to be painful before injury, like pricking the skin with a needle [49]. In this study, we used an electronic von Frey apparatus with a rigid plastic monofilament (punctate stimulation) rather than a series of filaments with different bending forces [50]. The EVF stimuli evoked a punctate withdrawal response before injury, and the animals’ withdrawal responses were exaggerated after injury, as less force of the monofilament was needed for the withdrawal response to be elicited. In addition, guarding behavior, paw-shaking and grooming of the CFA-injected paw was occasionally observed following stimulation. Therefore, the lower thresholds seen in this study were considered to be hyperalgesia-related. Further, the pain response was provoked by a stimulation of the plantar surface of the paw, a test-site remote from the injury site. Therefore, the lowered mechanical thresholds measured in this study could possibly be due to of secondary hyperalgesia arising from central changes, reflecting the peripheral input on the central nervous system (CNS) [51]. In some previous studies, secondary hyperalgesia has also been measured in the contralateral hind paw to an injury (reflex neurogenic inflammation) [52–55]. However, contralateral effects were not detected in this study. In fact, contralateral paws of CFA-injected rats showed a higher tolerance to pressure compared to the control group. This suggests a shift in weight bearing onto the contralateral hind limb, compensating the ipsilateral CFA-injected hind limb. Using other objective methods to test the effectiveness of analgesia may be of higher translational value than EVF testing in this model.

In an attempt to detect and quantify spontaneous pain, the rat grimace scale was applied. The RGS scores were low for all groups (below a score of 1) suggesting mild pain intensity. Differences between groups were detected in the treatment-period (day 1–15). Possible positive treatment effects were seen, as the carprofen and buprenorphine treated groups were significantly lower in scores compared to the vehicle group, with the buprenorphine group having the lowest scores. In addition, the buprenorphine group was not significantly different from the control group, unlike the carprofen group. In this study, the action units were evaluated when the rats were in their home cages, after the lid was lifted off in the presence of an observer. It can be argued that these conditions were not optimal, because animal behaviour changes in the presence of an observer (observer effect), the observer is able to observe the whole body and not just the head (observer bias), and there is an overall lower accuracy from not having video-playback [42]. However, approaches of assessing images or videos is however far more time-consuming and a given intervention can only be evaluated retrospectively this way, as opposed to being able to assess the animal’s pain as it occurs. Multiple factors can affect the RGS outcome and there are studies suggesting that the RGS publication record involves publication bias, and that it may not be applicable to all studies [56].

It is well known that almost all human contact with rodents, such as handling and restraint or anaesthesia, is stressful to the animal. This results in an increased release of corticosterone by the adrenal cortex into the blood stream and subsequent excretion in urine and faeces [57–59]. Corticosterone levels have been used as a biomarker of stress, and indirectly of pain, in several species [20, 60–62]. Despite that the FCM scores in general were low in the present study, all CFA groups demonstrated an increase in scores post-injury compared to pre-injury. Improvement of scores were detected in both treatment groups in the treatment period, with the buprenorphine group showing the greatest improvement of scores compared to the untreated group. The rise in FCM at Day 15 in the control group is mainly due to that one single rat had doubled the amount of FCM at that time point. It is thus not a systematic increase, and the increase is only apparent, not statistically significant. We do not have any particular explanation for this—if that rat was particularly stressed at that point or if it could be an error in the quantification process.

All CFA-injected animals displayed severe arthritic pathology, involving inflammatory granulomatous lesions intraarticular and periarticular to the tibio-tarsal joint, as expected. However, the formation of granulation tissue within the synovial subintimal region resulted in hypertrophic synovial tissue expanded to affect several other joints in the ankle. In this study and a previous study [30], we used a CFA volume of 20 μL to induce TTJ monoarthritis, which is a smaller volume, compared to many other studies [40, 63–66]. Even with a volume of 20 μl, unintended massive inflammation affected surrounding tissue in the ankle. Performing the induction into a bigger and isolated joint, such as the knee joint, could despite other complications, be an advantage. The use of smaller volumes of CFA will also be investigated in the future.

A relatively long-term treatment-period seen in this study, may be unnecessary in this model, especially in relation to possible treatment-related long-term adverse effects, e.g. body weight decreases upon buprenorphine treatment as indicated in this study, or opioid tolerance that has been demonstrated in other studies [67, 68]. A shorter treatment-period within the first week, may be more desirable, avoiding possible adverse effects of treatment. However, the study was designed to show that even with the use of long-term buprenorphine treatment, we managed to develop a relevant model with enhanced animal wellbeing and the desirable histopathological changes of monoarthritis. Therefore, the authors encourages the use of analgesia initially unless there is a scientific justification otherwise. However, we cannot exclude molecular changes after buprenorphine treatment in the post-induction period—both in the up/down regulation of opioid receptors and in the wind-up/development of the central sensitization etc. and more experiments are required to explore this further.

Findings in this study provide no immediate reason to withhold buprenorphine analgesia to alleviate unnecessary pain in the complete Freund’s adjuvant-induced monoarthritis rat model, in the investigated time window. Buprenorphine treatment resulted in some improvement of facial pain expression scores, mobility, stance and lameness scores, and it did not suppress the CFA-induced ankle swelling, unlike carprofen treatment. Proper development of CFA-induced arthritis was confirmed in all buprenorphine treated rats, evaluated histologically, although it should be pointed out that quantitative analysis of the histopathology is needed in future studies to clearly validate differences between treatment groups. Our study highlights the importance of a multimodal-behavioural approach in the assessment of pain and effects of analgesia. While the current study explored an elaborate long-term analgesia regimen, which may not be necessary for standard experiments, greater attention to alleviating pain during the first three days post-induction, where the well-being appears to be most compromised, should be paid in future studies. Lastly, induction of the model could be further optimized with regards to confining the inflammation to the joint, avoiding spread to the surrounding tissue. Whilst further studies are needed to elucidate if post-induction analgesia produces neurobiological changes that may affect the use of the model for specific experiments, we see no reason for not providing at least a minimal level of analgesia in the immediate post-induction days to improve the animal welfare.

## Supporting information

S1 Dataset All data obtained during this study. (XLSX)

S1 Raw images (DOCX)
