# Supplementary figures and images for: Effects of buprenorphine on model development in an adjuvant-induced monoarthritis rat model
Source: PLoS One. 2022 Jan 13;17(1):e0260356. doi: 10.1371/journal.pone.0260356 (PMC8757907; doi:10.1371/journal.pone.0260356)

**Fig 1**

**Fig 2**

**Fig 3**

**Fig 4**

**Fig 5**

**Fig 6**

**Fig 7**

**Fig 8**


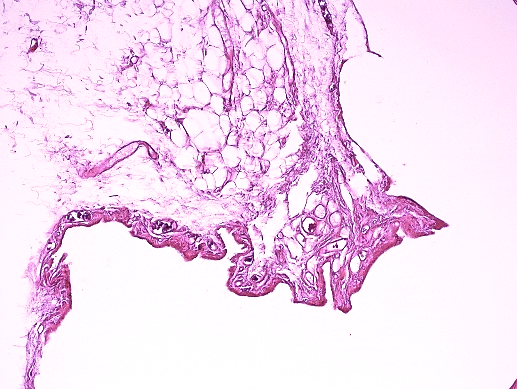

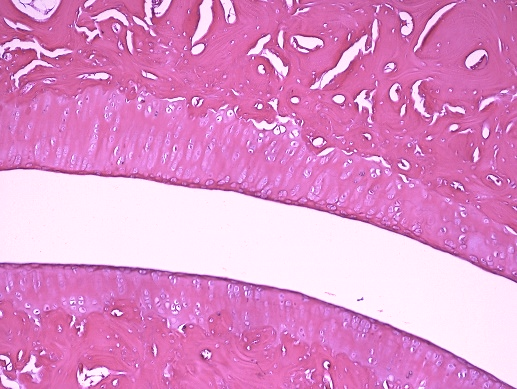

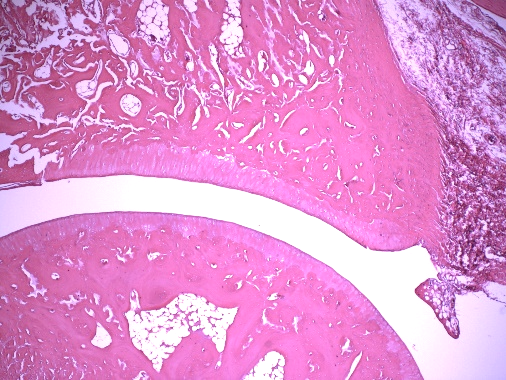


**t**

**C**

**B**

**A**

**tb**

**b**

**c**

**s**


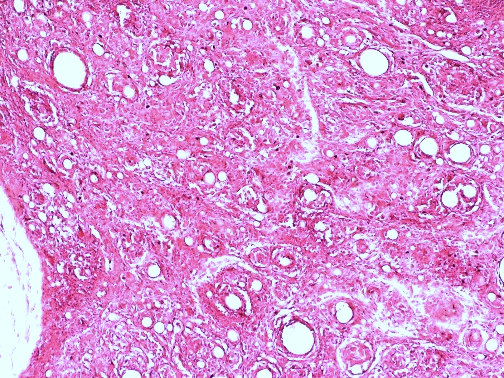

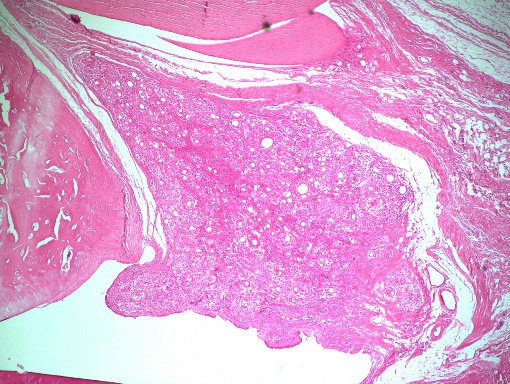

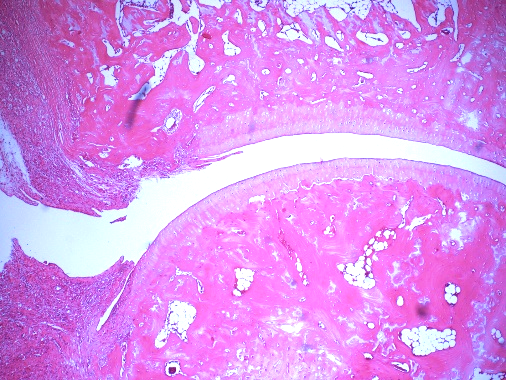


**t**

**F**

**E**

**D**

**tb**

**s**

**s**

Supplement: S1 Raw images — (DOCX) [file pone.0260356.s002.docx]
